# Preliminary Assessment of an Ambulatory Device Dedicated to Upper Airway Muscle Training in Patients With Sleep Apnea: Proof-of-Concept Study

**DOI:** 10.2196/51901

**Published:** 2024-04-15

**Authors:** Patrice Roberge, Jean Ruel, André Bégin-Drolet, Jean Lemay, Simon Gakwaya, Jean-François Masse, Frédéric Sériès

**Affiliations:** 1 Mechanical Engineering Department Université Laval Quebec City, QC Canada; 2 Centre de recherche Institut Universitaire de Cardiologie et de Pneumologie de Québec Université Laval Quebec City, QC Canada

**Keywords:** obstructive sleep apnea/hypopnea syndrome, OSAHS, myofunctional therapy, myotherapy, oral, orofacial, myology, musculature, labial, buccal, lingual, speech therapy, physiotherapy, physical therapy, oropharyngeal exercises, oropharyngeal, pharyngeal, pharynx, hypopnea, lip, home-based, portable device, devices, ambulatory, portable, monitoring, apnea, mouth, lips, tongue, facial, exercise, exercises, myofunctional, continuous monitoring, sleep-disordered breathing, sleep, breathing, tongue exercise, lip exercise, mHealth, muscle, muscles, muscular, airway, sleep apnea

## Abstract

**Background:**

Obstructive sleep apnea/hypopnea syndrome (OSAHS) is a prevalent condition affecting a substantial portion of the global population, with its prevalence increasing over the past 2 decades. OSAHS is characterized by recurrent upper airway (UA) closure during sleep, leading to significant impacts on quality of life and heightened cardiovascular and metabolic morbidity. Despite continuous positive airway pressure (CPAP) being the gold standard treatment, patient adherence remains suboptimal due to various factors, such as discomfort, side effects, and treatment unacceptability.

**Objective:**

Considering the challenges associated with CPAP adherence, an alternative approach targeting the UA muscles through myofunctional therapy was explored. This noninvasive intervention involves exercises of the lips, tongue, or both to improve oropharyngeal functions and mitigate the severity of OSAHS. With the goal of developing a portable device for home-based myofunctional therapy with continuous monitoring of exercise performance and adherence, the primary outcome of this study was the degree of completion and adherence to a 4-week training session.

**Methods:**

This proof-of-concept study focused on a portable device that was designed to facilitate tongue and lip myofunctional therapy and enable precise monitoring of exercise performance and adherence. A clinical study was conducted to assess the effectiveness of this program in improving sleep-disordered breathing. Participants were instructed to perform tongue protrusion, lip pressure, and controlled breathing as part of various tasks 6 times a week for 4 weeks, with each session lasting approximately 35 minutes.

**Results:**

Ten participants were enrolled in the study (n=8 male; mean age 48, SD 22 years; mean BMI 29.3, SD 3.5 kg/m^2^; mean apnea-hypopnea index [AHI] 20.7, SD 17.8/hour). Among the 8 participants who completed the 4-week program, the overall compliance rate was 91% (175/192 sessions). For the tongue exercise, the success rate increased from 66% (211/320 exercises; SD 18%) on the first day to 85% (272/320 exercises; SD 17%) on the last day (*P*=.05). AHI did not change significantly after completion of training but a noteworthy correlation between successful lip exercise improvement and AHI reduction in the supine position was observed (*R*_s_=–0.76; *P*=.03). These findings demonstrate the potential of the device for accurately monitoring participants’ performance in lip and tongue pressure exercises during myofunctional therapy. The diversity of the training program (it mixed exercises mixed training games), its ability to provide direct feedback for each exercise to the participants, and the easy measurement of treatment adherence are major strengths of our training program.

**Conclusions:**

The study’s portable device for home-based myofunctional therapy shows promise as a noninvasive alternative for reducing the severity of OSAHS, with a notable correlation between successful lip exercise improvement and AHI reduction, warranting further development and investigation.

## Introduction

Obstructive sleep apnea/hypopnea syndrome (OSAHS) is a common condition that affects a large portion of the world population [1]. It is estimated that mild to severe OSAHS affects 24% of men and 9% of women in North America [2], with an increase in prevalence over the last 2 decades [3]. OSAHS originates from repetitive closure of the upper airway (UA). The negative impacts of OSAHS include a deterioration of quality of life [4] and an increase in cardiovascular and metabolic morbidity [5-10]. Currently, the gold standard for treatment of this condition is continuous positive airway pressure (CPAP) [5] machines, which provide constant pressure to the sleeping patient via an oral or nasal mask. While this method has proven to be effective in reducing the adverse effects of OSAHS, it has been reported that from 46% to 83% of patients do not adhere to the treatment [11]. The causes of this low adherence rate may include treatment unacceptability, general discomfort, side effects (mask leaks, pressure intolerance, skin irritation, mouth dryness), bed partner intolerance, or a combination of these causes [12].

Alternatively, therapies targeting the UA muscles have been developed to decrease the disease severity [13-15]. Myofunctional therapy is a noninvasive approach in which patients are tasked with exercises of the lips, tongue, or both to target oropharyngeal functions [16]. It has been observed that myofunctional therapy may decrease the apnea-hypopnea index (AHI) by 50% in adults and by 62% in children [16]. For myofunctional therapy to be effective, the patient must perform the exercises daily. However, monitoring the quality and frequency of the exercises is pivotal to supporting implantation of such treatment and may be challenging outside of the laboratory setting. Therefore, there is a need for developing a home reeducation setup where patients can perform daily exercises with continuous monitoring of program adherence and exercise performance.

We developed a portable device that allows completion of tongue and lip myofunctional therapy while providing precise performance monitoring of performance and adherence to exercise. The aims of this clinical study were to evaluate task performance and treatment adherence to a 4-week training session and its efficacy in improving sleep-disordered breathing.

## Methods

### Study Design

Ten patients with untreated mild or moderate OSAHS who were referred to our sleep clinic volunteered to participate in this study. These patients were men and postmenopausal women aged ≤65 years who had a BMI ≤30 kg/m^2^ and regular sleep habits free of sleep debt (caused by, eg, insomnia or sleep deprivation). Their initial OSAHS diagnosis and severity were established by conventional sleep studies (level 1 or 3) performed at our local sleep clinic. Consecutive patients fulfilling the entry criteria were offered enrollment, and recruitment was completed within 6 months. A polysomnographic study (level 2, Embla Titanium; Natus) and the Epworth Sleepiness Scale (ESS) were completed just before and after a 4-week training program. A registered sleep technician who was blind to the protocol performed polysomnography scoring according to standard American Academy of Sleep Medicine criteria [17]. The participants were asked to perform the full training (35 minutes) 6 days a week for 4 weeks. The first session was completed in our research laboratory and the remainder were done at the participant’s home. A follow-up was completed by phone on the first and third home training days during the first week and once a week thereafter.

### Ethical Considerations

The Ethics Review Board of Institut Universitaire de Cardiologie et de Pneumologie de Québec approved the protocol (2020-3246), which conformed to the guidelines set forth by the Declaration of Helsinki, and written informed consent was obtained from all participants.

### Module Overview

**Figure 1:**
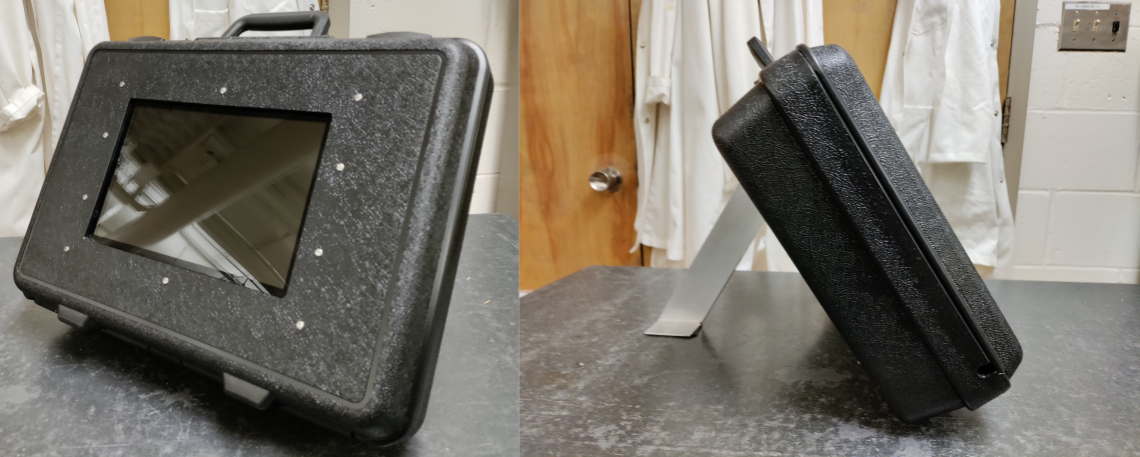
Pictures of the module packaged in a customized briefcase with a touch screen, computing unit, and hardware.

A briefcase-sized module that can precisely monitor lip and tongue pressure with a custom-made mouthpiece is presented in this paper. The module presented in Figure 1 is composed of 3 main parts: the mouthpiece, the interface electronics, and the user interface. The mouthpiece design is based on a 3D scan of the patient’s teeth and has 2 separate embedded internal cavities to record lip and tongue pressure. The mouthpiece is made out of silicone cast in 3D-printed sugar molds. The pressure developed by contraction of the lips or tongue is read from inside the mouthpiece with transducers and transferred to a computing unit. The module includes a touch screen with an intuitive interface for user interaction. The software combines precise pressure measurements with user calibration and engaging games to maximize therapy adherence. The training presented in the software is based on the tongue-protrusion task program presented by Svensson et al [18], in which the participants are asked to exert a force with their tongue on a force transducer and maintain a certain level of force for 1.5 seconds before releasing. The module is packaged in a customized briefcase for a robust and easy-to-transport solution.

### Mouthpiece Design

**Figure 2:**
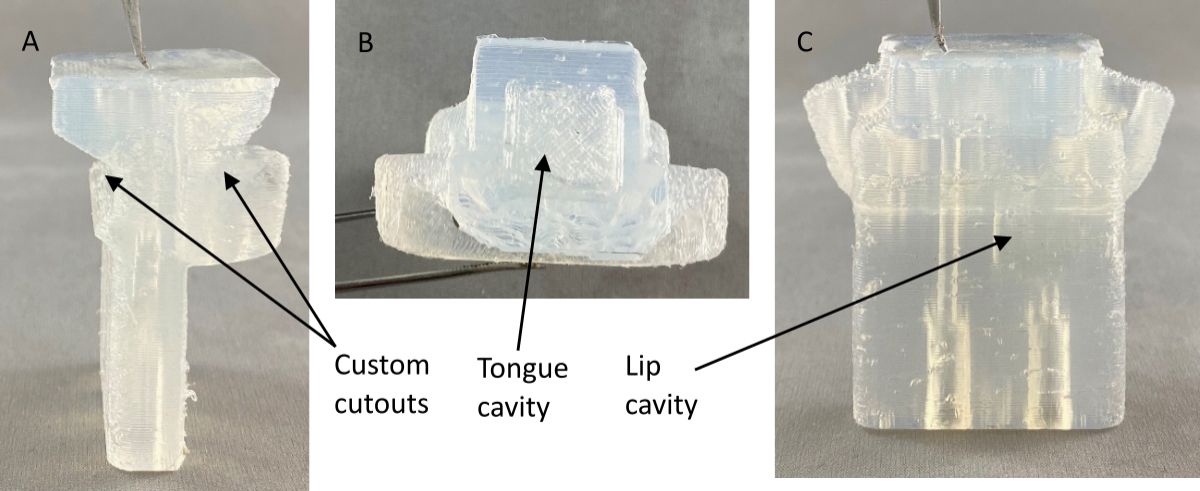
Images of the custom mouthpiece. (A) Side view; (B) top view; and (C) front view.

To maximize adherence to the treatment, a custom mouthpiece was made for each participant. A 3D scan was made of a participant’s teeth by a dentist, and the dental print was digitally removed from a mouthpiece template using Meshmixer (version 3.5.474; Autodesk). Having a custom cutout allowed the mouthpiece to clamp naturally onto the patient’s teeth and gums, as presented in Figure 2A. The mouthpiece design includes 2 distinct internal cavities acting as pressure chambers, one for the lips and one for the tongue, as presented in Figures 2B and 2C. The lip cavity is located in the front part of the mouthpiece where the lips naturally rest. The cavity has a thin bottom and top wall where the pressure from the lips is applied. The second cavity is located in the back portion of the mouthpiece (behind the incisors) and has a thin back wall where the tip of the tongue is positioned during tongue exercises. Both cavities have tunnels connecting them to the front of the mouthpiece, where connectors can be installed to 2 distinct pressure transducers. The changes in cavity volume produced by thin wall deformation from lip or tongue movements increase the respective inner pressure.

From the digital model of the mouthpiece, a mold was created with sugar using a custom 3D printer and molding technique [19-21]. Silicon was poured into the mold, and air bubbles were removed in a vacuum chamber. Once the silicon solution solidified, the sugar was dissolved in water to free the mouthpiece.

### Hardware Description

**Figure 3:**
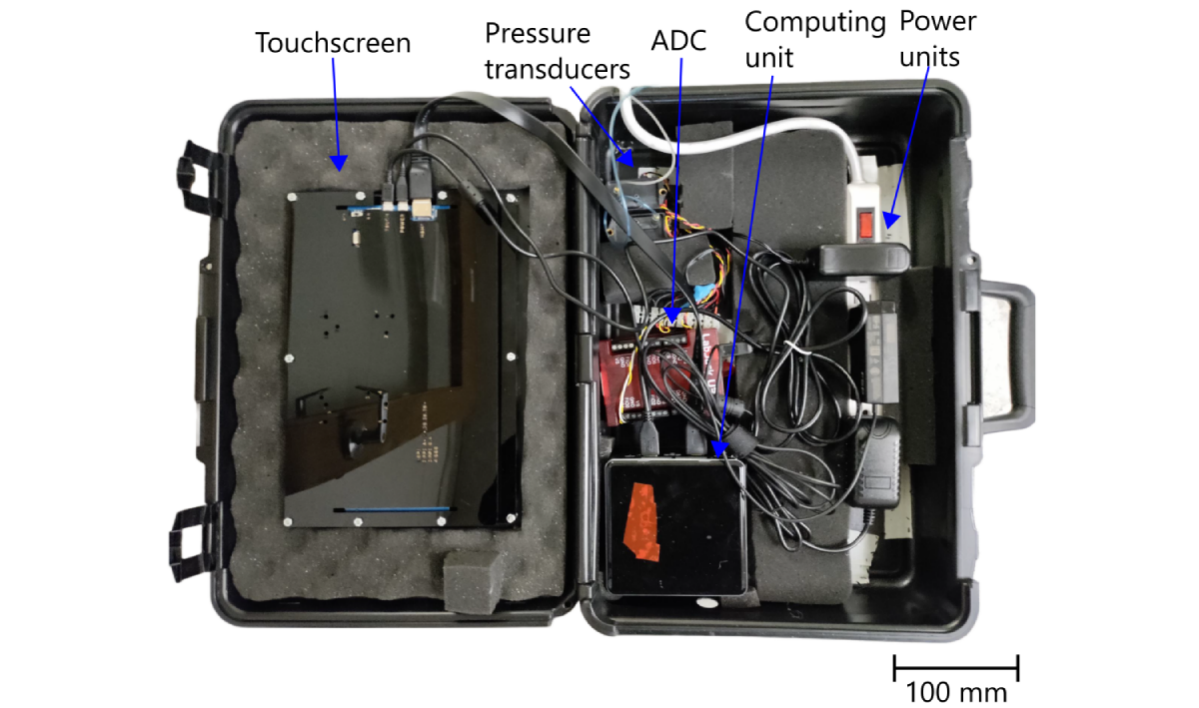
Picture of the different hardware components packaged inside the briefcase. ADC: analog-to-digital converter.

The absolute pressure in the lip and tongue cavities is linked to 2 pressure transducers (Omega PX142-002D5V) by EVA tubing and adaptors. The output voltage of the transducers is then adapted to maximize the operating range of the analog-to-digital converter (Labjack U3-LV). A computing unit (NUC7i7BNH) is used to read the values from the 2 converters. The user interacts with the software on the computing unit with a touch screen (Waveshare; this unit uses a 10.1-inch HDMI-connected LCD). All the described hardware, as well as power units, are packaged inside a briefcase, as shown in Figure 3.

### Software Description

**Figure 4:**
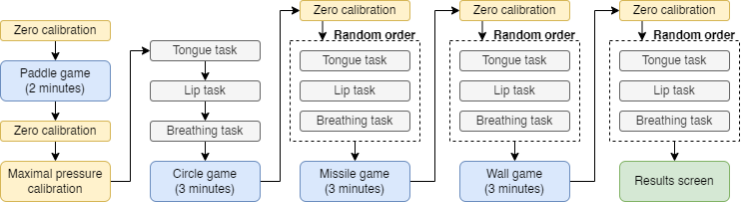
Flow chart describing the different steps of a training session.

The software comprises 3 main parts: initialization, training, and games. The participant must go through a sequence alternating between these 3 parts to complete the session. Figure 4 presents a flow chart describing the process of a training session.

#### Zero Calibration

Once the mouthpiece has been installed, an initialization step must be performed. The participant is asked to release pressure from the tongue and lips to measure the baseline values with the display (presented in Multimedia Appendix 1, Figure S1. This step is very important since the pressure in the cavities is also influenced by temperature variation. When the participant first puts the mouthpiece on, it will gradually heat up until a steady state is reached after approximately 1 minute. The first zero calibration is followed by a 2-minute game that does not require precision in the pressure measurements. This game allows the mouthpiece temperature to reach a steady-state level. A second zero calibration takes place at the end of the game. The zero calibration step is automatically repeated before each training session to ensure that no new temperature offset affects the pressure measurements.

#### Maximal Pressure Calibration

After the first zero calibration and the warm-up game, the participant is asked to set a baseline for both tongue protrusion and lip contraction maximal pressure. The participant starts on a page with a vertical bar corresponding to the actual developed tongue pressure, as presented in Multimedia Appendix 1, Figure S2. The participant is asked to develop maximal tongue pressure before releasing and then press OK in 3 consecutive attempts. If the absolute deviation divided by the mean value of the maximal value is greater than 7.5%, the process is repeated; otherwise, the mean value is used as the baseline maximal tongue pressure. Subsequently, the same steps are completed for the lips.

#### Training

The training section consists of 3 tasks targeting, respectively, the tongue, the lips, and control of breathing. The tongue and lip pressure tasks are based on the work of Svensson et al [18]. The display includes a white background, a green box, and an orange square, as presented in Multimedia Appendix 1, Figure S3. The orange square displays the pressure level applied to the corresponding cavity. The participant is tasked with placing the orange square into the green box. The cycle starts with the green box at the bottom for the first 8 seconds, and the participant is asked to release the pressure from the cavity. The green box then rises to a level of pressure between 3% and 5% of the maximal pressure for 3 seconds. The first 1.5 seconds allow the user to react and adjust muscle contraction to fit within the targeted pressure range. A success score corresponding to the percentage of time the participant successfully applied pressure in the given range is computed during the last 1.5 seconds. The task repeats for 10 cycles, after which a cumulative score is computed.

For the breathing task, the participant is asked to continuously apply a small lip pressure of 4% of the maximal value. Visual feedback on the lip pressure is given to the user via a vertical bar. The participant is tasked with following a breathing pattern indicated on the screen. This task includes 7 breathing cycles, with 8 seconds for inhaling and 8 seconds for exhaling. The success of this task is defined by the fraction of the total time when the user has applied sufficient pressure. The user progression during this exercise is displayed with a horizontal progress bar, as presented in Multimedia Appendix 1, Figure S4.

#### Games

The games are intended as relaxing activities between the formal metered training exercises described above. The first game is a paddle and ball game where a ball bounces off the top and bottom walls as well as paddles on the side. The user can move the paddles up in proportion to the pressure applied to the lip (blue paddle) and tongue (green paddle) cavities, respectively. The objective is to prevent the ball from hitting the edges behind the paddles. Every time the ball bounces off the paddle, a counter is incremented and the ball speeds up. The counter resets once the ball hits the edge behind a paddle. The user can track the high score of the current session on the screen, as presented in Multimedia Appendix 1, Figure S5.

The second game is called the circle game. The user controls the position of a point; lip pressure controls the horizontal position and tongue pressure controls the vertical position. A yellow circle appears on the screen, and the user must combine lip and tongue pressure to place the point inside the circle. The circle turns green once the point is within its radius and must remain green for 3 seconds to succeed. Subsequently, the circle shrinks and appears at a new position. The user has 30 seconds to place the point steadily in the circle before the score resets; 7 seconds are added to the timer after each success. The current session’s high score is also displayed on-screen. The circle game display is presented in Multimedia Appendix 1, Figure S6.

In the missile game, the user must apply a small degree of tongue pressure and then release it to send a colored missile. The missile travels upward to a circle with 6 equal sections of different colors. Changing lip pressure allows the user to rotate the circle so that when the missile touches the circle, it collides with the section with the matching color. For each success, the missile velocity increases. The missile game display is presented in Multimedia Appendix 1, Figure S7.

The fourth game is the wall game, in which a ball moves horizontally toward a wall with a hole. The user must move the hole to let the ball through. The hole moves up incrementally when the user presses then releases lip pressure and moves down in the same way with tongue pressure. After each success, a new ball appears at a new height with a greater speed. The current and high scores are displayed on-screen, as presented in Multimedia Appendix 1, Figure S8.

#### Results Screen

Once the user goes through the full training session (ie, 4 breathing tasks, 4 lip tasks, 4 tongue tasks, and 4 games), the results of each task are presented as a bar graph and are archived in a file.

### Outcomes

The primary outcome of this study was the degree of completion of the exercises and adherence to treatment. The secondary outcomes were changes in sleep and breathing variables following training.

Compliance was defined as the number of completed sessions during the 4 weeks divided by 24 (6 sessions per week for 4 weeks). Tongue, lip, and breathing exercise success rates were defined by the percentage of time the participant successfully applied pressure in the given range. Results are presented with a 96% CI (1.96 SD). A mixed model was defined using a random intercept for the analysis of the changes in anthropometric data, the ESS score, sleep data, and the exercise success rate. One factor was associated to the before-and-after-intervention comparison. As the data were correlated, the normality assumption was verified with the Shapiro-Wilk test using residuals from the statistical model and transformed with the Cholesky metric. The graphical representation of marginal linear predictors with studentized residuals suggests the homogeneity of variances. Statistical significance was defined as a 2-tailed *P*<.05. Associations between AHI and adherence, as well as success rates, were assessed with Spearman correlations. Analyses were performed using SAS (version 9.4; SAS Institute).

## Results

The characteristics of our study population are displayed in Table 1. This study included 2 female participants. Breathing disturbances were mostly of moderate severity, except for 1 participant who had severe sleep apnea documented during the pretraining home sleep recording. A total of 2 of the 10 recruited participants did not complete the 4-week training program due to a lack of motivation. The remainder of the participants successfully completed at least 75% (18/24) of all sessions, as presented in Table 2, with an average compliance of 91% (175/192).

For the tongue exercise, the success rate increased from 66% (211/320; SD 18%) on the first day to 85% (272/320; SD 17%) on the last day (*P*=.05). For the lip exercise, it increased from 78% (248/320; SD 18%) on the first day to 87% (278/320; SD 16%) on the last day (*P*=.25), as presented in Table 3. It is important to note that for both exercises the success score decreased in participant 2 while it improved for all other participants, except for the lip exercise for participant 7. The success rate of the breathing exercise increased from 86% (275/320; SD 24%) the first day to 96% (307/320; SD 10%) on the last day (*P*=.24).

**Table 1:** Anthropometric, symptom, and sleep characteristics before and after 4 weeks.

	Pretraining value, mean (96% CI)	Posttraining value, mean (96% CI)	*P* value
Age (y)	48 (26 to 70)	N/A^a^	N/A
BMI (kg/m^2^)	29.3 (25.8 to 32.8)	29.3 (26.0 to 32.6)	.73
ESS^b^ score	11.3 (–0.1 to 22.7)	9.5 (–1.7 to 20.7)	.23
AHI^c^ (events/h)	20.7 (2.9 to 38.5)	17.7 (–3.5 to 38.9)	.10
AHI supine (events/h)	33.4 (–3.4 to 70.2)	26.7 (–1.6 to 54.0)	.37
AHI RMI^d^ (events/h)	25.5 (–6.1 to 57.1)	23.3 (–15.1 to 61.7)	.49
ODI^e^ (events/h)	20.3 (–6.0 to 46.6)	19.2 (–9.0 to 47.4)	.66
Time supine (%)	43.7 (–19.0 to 106.4)	43.6 (–19.1 to 106.3)	.99
TST^f^ (minutes)	420 (347 to 493)	395 (268 to 522)	.17
TST <90% SaO_2_^g^ (%)	1.6 (–0.9 to 4.1)	1.7 (2.0 to 5.4)	.75

^a^N/A: not applicable.

^b^ESS: Epworth Sleepiness Scale.

^c^AHI: apnea-hypopnea index.

^d^RMI:respiratory mechanic instability.

^e^ODI: oxygen desaturation index.

^f^TST: total sleep time.

^g^SaO_2_: oxygen saturation of arterial blood.

**Table 2:** Number of training sessions completed during the 4-week training program.

Participant	Week 1	Week 2	Week 3	Week 4
1 (total=24)	6	6	6	6
2 (total=20)	5	4	5	6
3 (total=24)	6	6	6	6
4 (total=22)	6	5	5	6
5 (total=22)	6	6	6	4
6 (total=23)	6	6	6	5
7 (total=18)	4	3	6	5
8 (total=22)	6	4	6	6

**Table 3:** Success rate at baseline and after 4 weeks of training.

Participant	Tongue success rate, %		Lip success rate, %	
	Baseline	Posttraining	Baseline	Posttraining
1	34	82	38	80
2	73	47	78	51
3	61	99	90	95
4	53	79	69	89
5	62	90	92	97
6	73	95	80	97
7	92	95	91	90
8	79	92	88	95

During the study period, there was no significant change in BMI, ESS score, AHI, and other polysomnography-derived parameters, as displayed in Table 1. Table 4 presents the variation in AHI before and after the 4-week training for each participant. It illustrates that the index of each participant improved, with the exception of participants 2 and 3.

A significant correlation was found between the decrease in AHI in the supine position and the change in success rate for the lip exercise (*R*_s_=–0.76; *P*=.03).

**Table 4:** Effects of 1 month of training on obstructive sleep apnea/hypopnea syndrome severity.

Participant	Pretraining AHI^a^, events/h	Posttraining AHI, events/h
1	13.6	11.6
2	15.4	19.6
3	41.0	40.8
4	17.4	6.3
5	25.0	21.3
6	20.5	14.9
7	12.6	8.3
8	20.2	19.0

^a^AHI: apnea-hypopnea index.

## Discussion

### Principal Findings

The results of this study illustrate the feasibility of performing a training task focusing on the recruitment of different UA muscles while collecting major information such as adherence to the training program and objective measurements of task completion and success.

### Comparison to Prior Work

Myofunctional therapy is a relatively new treatment for sleep-disordered breathing and is based on a combination of regular exercises aiming at enhancing muscle recruitment from various oral and oropharyngeal structures [13]. Although its effect on AHI and sleep apnea–related symptoms has been shown in an increasing number of studies [16], the main challenge to its success remains the objective assessment of program adherence and exercise performance [22]. Of the 10 participants initially recruited, 8 successfully completed more than 75% (18/24) of the total number of sessions over a period of 4 weeks. Two of the initially recruited participants left the study less than 3 days after the start of the training. In comparison, rates of participant adherence reported in recent studies using myofunctional therapy with a mobile app were 75%, 90%, and 65% (15 minutes per session, 5 times/week for 3 months) [23-25]. In addition, Kim et al [26] found that in a myofunctional therapy support program with the help of exercise diaries, the reported adherence was 82%. In studies from Kim et al [26] and O’Connor et al [23-25], one could note the strong encouragement given to the patients through easy access to a health professional, encouraging text messages, or the use of a mobile app. In this study, a higher adherence rate was observed compared to the aforementioned studies. One potential explanation for this improvement is that the exercises were designed to induce motivation with games and visual feedback. Recently, a mobile app has been developed for this purpose that only requires a smartphone [24]. It first teaches the patient how to perform the exercise and then provides timely feedback on their performance. The results are saved over time, and the app promotes assiduity. It was observed that after 3 months, 75% of the patients completed the training at least 5 days a week [24] and the AHI of patients who adhered to the treatment decreased by 53.4% [23]. However, the quality tracking of the exercises is limited by the functionality of the smartphone and requires covering the screen with cling film or hypoallergenic plastic wrap every session, since the tongue touches the screen [24]. The proposed custom mouthpiece introduced in this paper enables more accurate measurement of lip and tongue pressure, ensuring enhanced exercise quality.

### Strengths

The multidimensional nature (exercises mixed with various training games) of the training program, its ability to immediately provide performance results for each exercise to the participants, and the measurement of treatment adherence are important strengths of our training program. As myofunctional therapy is based on an integrative approach, it is not possible to define which of the exercises may contribute most significantly to treatment success [13]. In recent studies, a combination of 9 exercises has been found to be sufficient to significantly decrease AHI [23,24]. Previous studies have focused on a single exercise and have found variable success [14,27]. Here, we used 3 exercises as a compromise between recruiting more of the muscles involved in the pathogenesis of OSAHS and keeping the workload at an acceptable level for participant motivation. Our results seem to show that a greater number of participants should have been recruited in order to see a higher impact on AHI reduction. Based on the results of our previous study [14], the training program duration was set at 4 weeks. This may have affected the results, as clear benefits in previously published myofunctional therapy studies were observed after 3 months of training. This difference may have helped to obtain a higher adherence rate while limiting the AHI reduction.

### Limitations

There were 3 limitations of this study. The first was the success rate. This 4-week program did not significantly influence OSAHS severity to the degree that we previously found with an intensive in-lab tongue-protrusion training session that lasted 1 hour in 1 week [14]. However, it should be emphasized that although AHI did not decrease significantly, a correlation was found between the increase in success rate for the lip exercise and AHI decrease in the supine position. Therefore, one possible explanation for the modest decrease in AHI could be that the success rate of the present exercises was much higher than our previous in-lab trial (for both success rate during the first session and the rate of increase during the training period). Previously, it started from an average of 28% up to 65% for the last session. Similarly, an initial success rate of 25% was observed by Svensson et al [18] in a similar 1-week tongue-training program that was devised to increase corticomotor excitability. The high success rate in this study was likely mainly due to the adjustments that were made to the experimental set-up in order to make it ambulatory. However, in our success rate calculation, we did not take into account the results of the game sessions, which also involved a learning process and accounted for about half the duration of a training session. These games were designed mainly to boost the patients’ motivation to continue the program. Future exercise settings could be individually adjusted to adapt exercise targets to participants’ baseline UA performance, with the goal of improving the success rate over time.

The second limitation was the selection of participants. Since it is not known to what extent anatomical UA abnormalities contribute to training program efficacy, no such selection criteria were used for our study population. It could be interesting to complete further studies focusing on patients with limited anatomical abnormalities according to practical clinical scores (ie, Mallampati and velopharyngeal scores). Apart from sleep apnea severity and degree of obesity, there were no selection criteria for participant selection. However, there may have been an indirect selection criterion due to the need for the participants to complete an additional preliminary visit at a dentist’s office to perform the 3D tooth scan. This may have interfered with individual willingness to enter into the trial. Identifying participants who will remain engaged with the training program is a crucial issue for such a treatment strategy. Having a training device available for demonstration in the setting of a sleep clinic could definitely help to identify participants who are likely to follow the requirements of a training program.

The third limitation was the sample size. Yet another explanation for the lack of an AHI decrease is that our sample size was affected by the dropout of 2 participants and by the increase in AHI observed after the intervention in 1 participant. This participant was the only one with a decreasing overall success rate. This particular patient did have difficulty remaining focused throughout the training month, probably due to excessive sleepiness. As OSAHS is a multifactorial disorder [28] in which anatomical and nonanatomical factors can interact to modulate the severity of the disease, patient selection may play an important role in the success of OSAHS muscle training. Therefore, for this particular participant, treatments targeting traits other than low muscle tone or function would have been effective for decreasing AHI and related symptoms.

### Conclusions

This study was an attempt to develop a prototype aimed at completing a simplified oral/oropharyngeal exercise program in an entertaining way in the comfort of a patient’s own home. The program gives the patient visual feedback, as well as the ability to monitor improvement. Patients were instructed to perform tongue protrusion exercises, lip pressure exercises, and controlled breathing in various playful tasks 6 times a week for 4 weeks. Session duration was about 35 minutes. While the AHI reduction was not significant, we found that the success rate for improvement in the lip exercise was correlated with AHI reduction in the supine position (*R*_s_=-0.76; *P*=.03). These results are a first steps toward the tuning of an ambulatory myofunctional therapy module able to accurately monitor the performance of participants in lip and tongue pressure exercises. This noninvasive approach may decrease the severity of OSAHS and represent an alternative to more invasive solutions, such as CPAP devices.

## Appendix

Multimedia Appendix 1 Screenshots of the software.

## Abbreviations

AHI: apnea-hypopnea index
CPAP: continuous positive airway pressure
ESS: Epworth Sleepiness Scale
OSAHS: obstructive sleep apnea/hypopnea syndrome
UA: upper airway

## Data Availability

The data sets generated during and/or analyzed during this study are available from the corresponding author on reasonable request.
