# Supplementary material for: Preliminary Assessment of an Ambulatory Device Dedicated to Upper Airway Muscle Training in Patients With Sleep Apnea: Proof-of-Concept Study
Source: JMIR Biomed Eng. 2024 Apr 15;9:e51901. doi: 10.2196/51901 (PMC11058550; doi:10.2196/51901)
Supplement: Multimedia Appendix 1 [file biomedeng_v9i1e51901_app1.docx]

## Multimedia Appendix A : Screenshots of the Software.


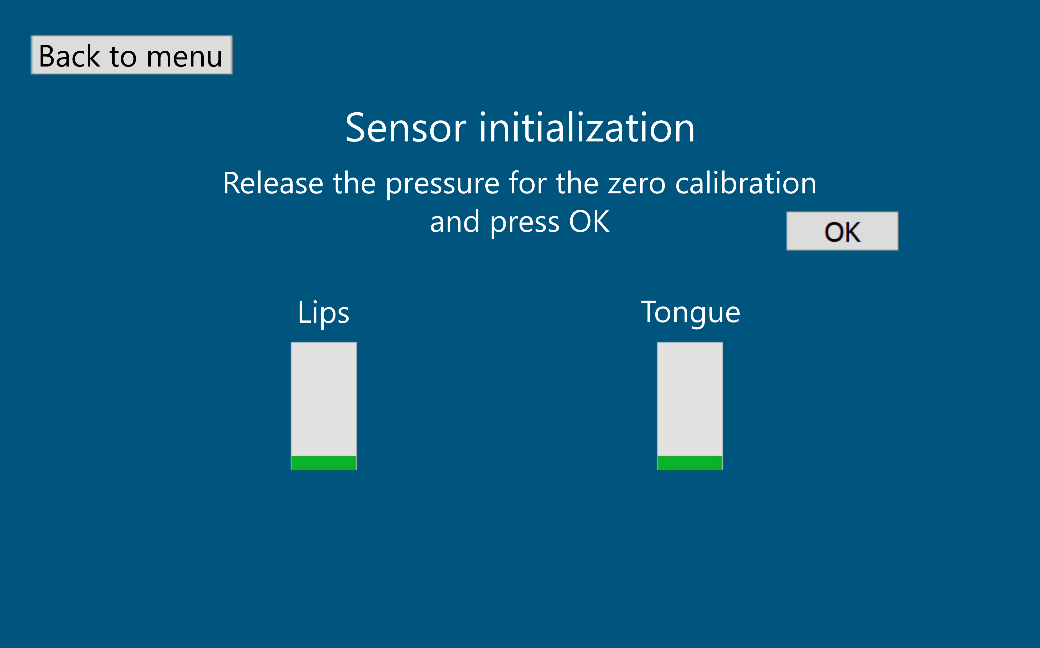


Figure S1: Screenshot of the zero calibration step of the software.


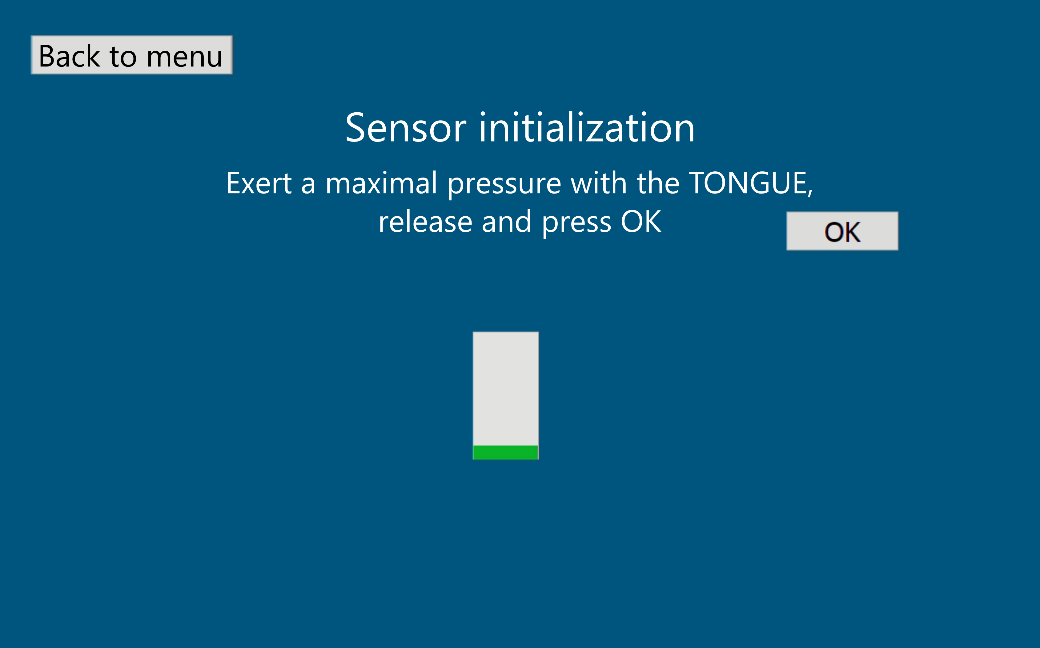


Figure S2: Screenshot of the maximal tongue pressure step of the software.


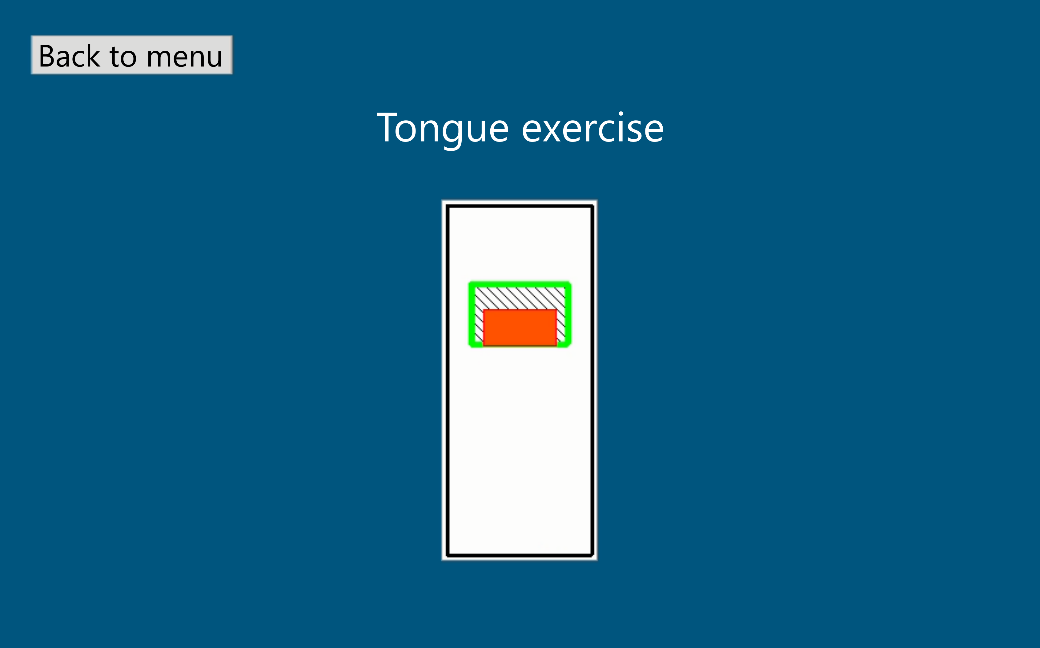


Figure S3: Screenshot of the tongue/lips exercise step of the software.


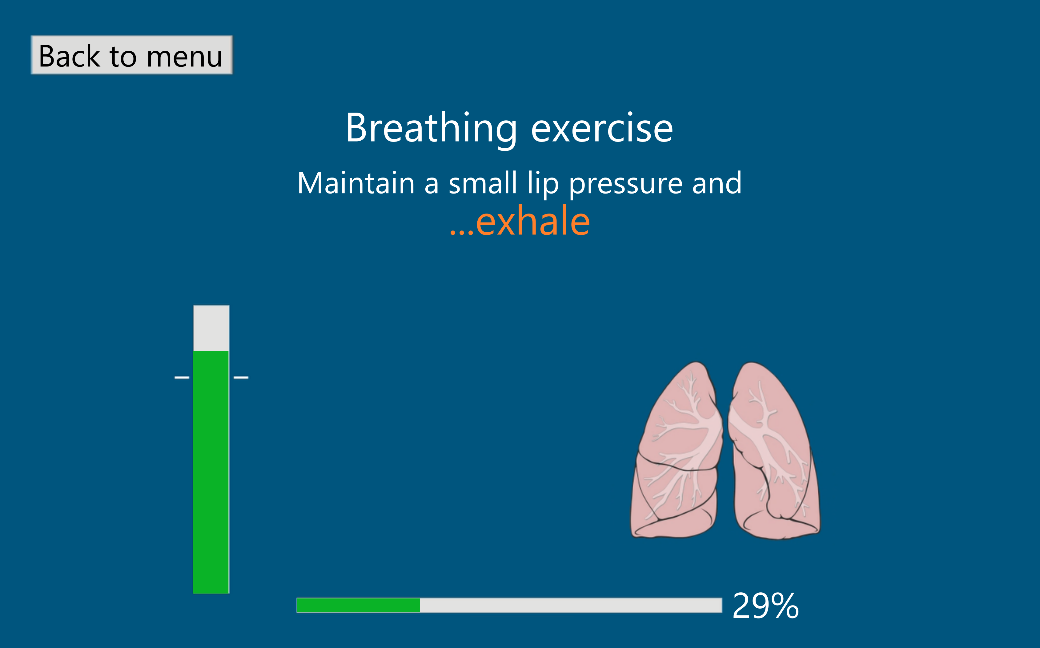


Figure S4: Screenshot of the breathing exercise step of the software.


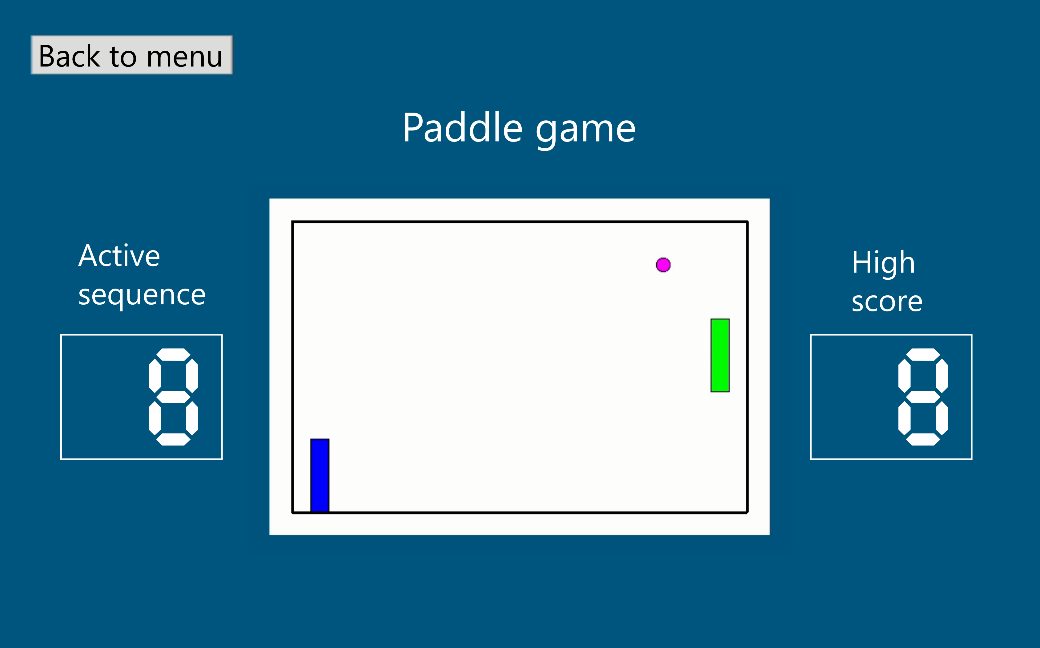


Figure S5: Screenshot of the paddle game step of the software.


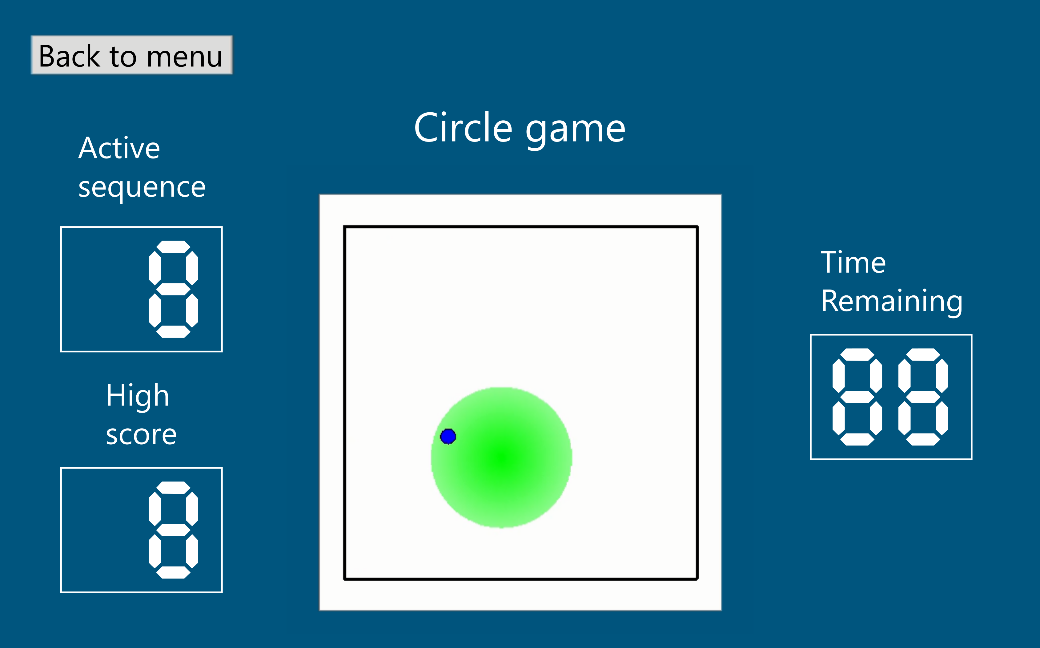


Figure S6: Screenshot of the circle game step of the software.


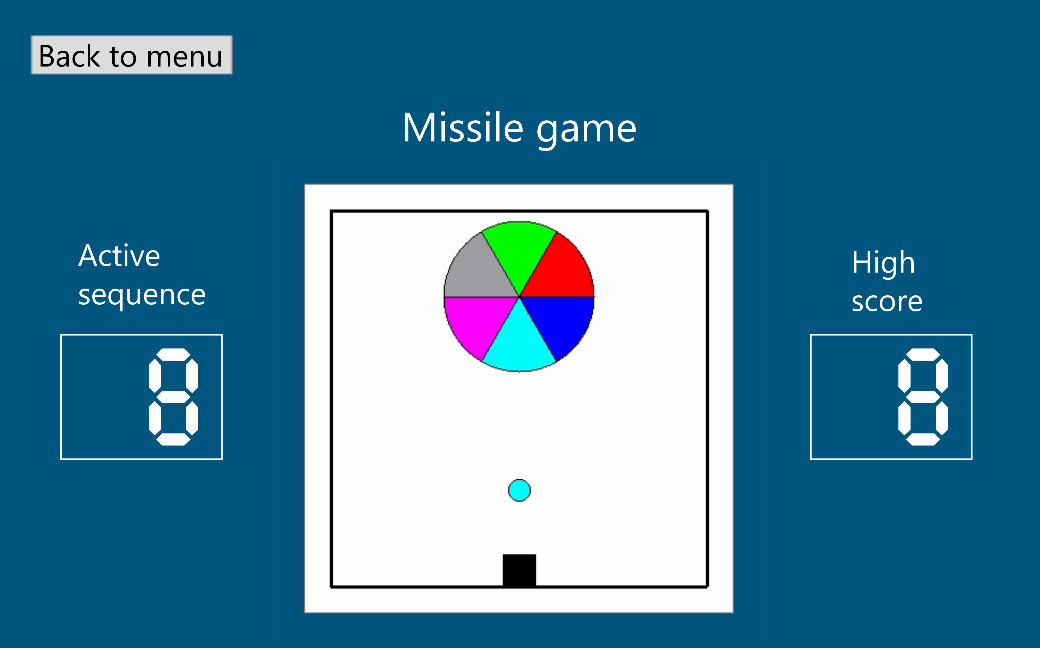


Figure S7: Screenshot of the missile game step of the software.


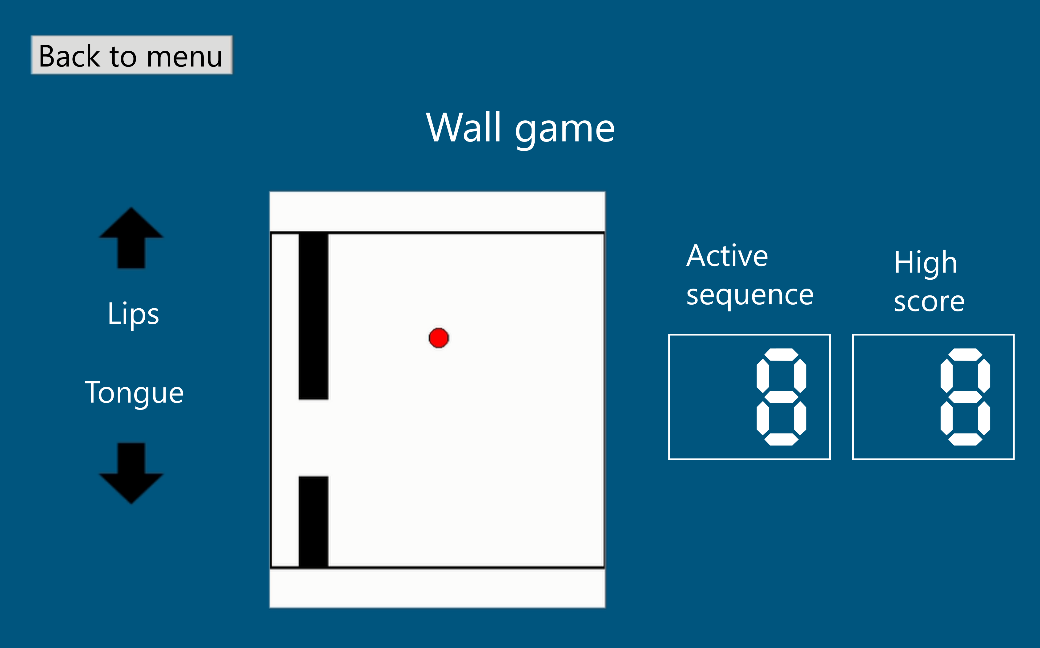


Figure S8: Screenshot of the wall game step of the software.
